# Supplementary material for: Are carbon emissions trading and green financial instruments synergistic? -Comprehensive quantitative research based on content analysis
Source: PLoS One. 2024 Mar 7;19(3):e0298601. doi: 10.1371/journal.pone.0298601 (PMC10919601; doi:10.1371/journal.pone.0298601)
Supplement: S1 Data — (ZIP) [file pone.0298601.s001.zip › Data/地级市Z_F工作报告.zip!/╔╜╬≈/╠1⁄2╘¡╩╨/└┤╫╘╣1⁄2╓┌║┼í╛┬φ┐╦╩2╛▌═°í┐_2005.docx]

各位代表：

现在，我代表市政府向大会作报告，请予审议，并请各位政协委员和其他列席人员提出意见。

一、2004年政府工作回顾

2004年是我市实现率先发展阶段性目标的决战之年。一年来，全市人民以“三个代表”重要思想为指导，认真贯彻党的十六届三中、四中全会精神，在省委、省政府和市委的正确领导下，全面落实科学发展观，团结一致，扎实工作，圆满完成了市十一届人大四次会议确定的各项任务，实现了率先发展阶段性目标。全年实现地区生产总值643.1亿元，增长15.7%；财政总收入120.2亿元，增长31.2%；规模以上工业增加值230.1亿元，增长24%；固定资产投资298.8亿元（城镇口径），增长51.4%；社会消费品零售总额226.3亿元，增长22.1%；城镇居民人均可支配收入9353元，增长13.2%；农民人均纯收入3873元，增长15.4%；城镇登记失业率3.4%；人口自然增长率4.5‰，空气质量二级以上天数224天。

结构调整成效显著。淘汰落后生产能力，取缔关闭土小企业和落后生产设施447个。深入开展焦化行业专项清理整顿，推平土焦坑2715个，清理已投产和在建项目57个，关闭小机焦12个，压缩生产规模123万吨。改造提升传统产业，更新改造投资增长50.3%，占固定资产投资的比重达到33.6%。放手发展新兴产业，国家级高新技术产业示范项目达到24个，占全省国家级高新技术项目的60%，在省会城市中居第4位。高新技术产业增加值占全部工业增加值的比重提高1个百分点，达到19%。不锈钢加工、电子信息、生物制药、新材料、精细化工等五大新兴产业增长43.8%，高于全市19.8个百分点。高新区、经济区、民营区工业增加值分别增长51.2%、93.1%和112%。不锈钢工业园入驻企业24户，实现产值9亿元。富士康工业园一期工程建成投产，二期进展顺利。个体私营企业纳税增长56%，民营经济占全市生产总值的比重提高3.6个百分点，达到38.9%。县区民营经济比重上升到80%以上，6户民营企业进入全国民营企业500强。

工业经济快速增长。规模以上工业企业实现利税83.5亿元，增长70%；工业经济效益综合指数131.6，提高26.9，创历史新高。大力开展“工业项目推进年”活动，扶持100个重点项目，太钢150万吨不锈钢、二电厂六期扩建项目开工建设，古交电厂一期进展顺利，新东方铝业二期即将投产，华宇重汽完成样车试制，蓝星TDI建成投产，一批技改项目陆续投产达效，对工业经济增长的贡献率达到50%。15户市属国有企业完成改制，14户改制工作进展顺利，县区属国有企业改制面90%以上。长城微光在香港创业板成功上市。

农村经济健康发展。认真落实中央1号文件精神，筹集资金6.3亿元，为农民办十件实事。在全市范围免征农业税1542万元，对种粮农民直接补贴402万元。种粮面积增加12.8%，粮食产量增长24.7%。粮食储备增加近一倍。农村经济总收入332亿元，增长12.3%。菜、肉、奶产量分别增长3%、6.8%和10.8%。农产品加工总产值33亿元，增长29.4%。乡镇企业增加值114亿元，增长36.6%；上缴税金18.9亿元，增长105.9%。解决了10万人的饮水困难和水质不达标问题，治理水土流失面积20万亩。完成村通油路工程743公里，98%的行政村通了油路、水泥路。完成10条207公里通乡油路改造，康西公路建成通车。对4.5万农村劳动力实施职业技能培训，转移就业4.3万人。3.1万农村贫困人口脱贫。

城市承载能力稳步提升。编制了不锈钢园区总体规划、民营区详细规划等重点规划，启动了新一轮城市总体规划修编工作。滨河东路北延、新城大街、学府街、新兰路、北涧河、玉门河治理、城南热源厂一期等12项重点工程按期竣工。滨河西路南北延伸、北郊污水厂扩建、垃圾焚烧发电等重点项目进展顺利。完成了和平北路等12条道路大修、70条小街小巷拓宽改造和100条小街小巷路灯改造，新建改建102个公厕，建成105个出租车候客专用通道。旧城改造和经济适用房开复工102万平方米，竣工40万平方米，房产交易24亿元。归集住房公积金12亿元，增长30%，累计达52.1亿元，为1.5万户职工发放了住房贷款。集中供热扩网512万平方米。新增绿地面积203公顷，城区绿化覆盖率37.6%，绿地率30.6%，人均绿地8.8平方米。城西水系荣获“中国人居环境范例奖”。加强水资源保护，关井压采23万吨/日。21个城中村改造试点完成控制性详细规划编制工作，9个村启动了住宅、环境改造工程。全力治理环境污染，三项污染指标平均下降17.2%。

内外贸易日趋繁荣。连锁经营、物流配送等新型流通业态蓬勃发展，新增超市、连锁便利店210家。对外贸易不断扩大，地区外贸出口总额26.3亿美元，增长73.3%。组织参加16项大型经贸活动，签约项目96个，引进资金43.6亿元人民币和6.7亿美元。新批外商投资企业27户，项目总投资3.9亿美元，增长48.7%。完成了拱极门、唱经楼、大关帝庙等一批文物景点的修缮保护，接待海内外游客1338万人次，旅游总收入51亿元，增长55.7%。

人民群众生活得到较好保障。城镇新增就业人数14.2万人，下岗失业人员再就业4.5万人，其中“4050”人员8751人，荣获“全国就业和再就业先进城市”称号。巩固“两个确保”，保障了3.1万名下岗职工和21.8万名离退休人员的生活，保障率和社会化发放率均保持100%。养老保险参保55.1万人，医疗保险参保70.5万人，农村养老保险参保9.5万人。低保标准由171元提高到183元，为9.2万名低保对象发放低保金9630万元，减免低保户水、暖、气等费用2079万元。建立“爱心救助超市”118个。为困难群众发放救济款747万元。残疾人就业培训工作进入全国先进行列。启动了廉租房制度，首批53户居民享受了优惠政策。清偿政府工程拖欠款5.32亿元，清偿拖欠农民工工资1.18亿元，清偿率100%。规范农村土地征用和城镇拆迁行为，妥善处理了一批拆迁居民安置遗留问题。

社会事业全面进步。建立了市级领导科技顾问制度。117个项目列入国家和省科技计划。开发高新技术产品50项，新建科技孵化基地5个，转化科技成果302项，专利成果转化率32%。完成了义务教育学制过渡和六周岁儿童入学工作，义务教育“一费制”全面实行。高考录取率61.7%，保持全省领先。改扩建中小学97所，改造中小学危房10.5万平方米。积极推进企业学校剥离，太铁、晋安、东方等16所学校移交工作基本完成，太钢、西山、太重、太化等71所学校移交正在进行。4所学校被认定为国家级重点中等职业学校。市属高校在校生达到2.8万人。中国晋阳文化艺术节、太原国际面食节等文化节会圆满成功。新闻出版市场进一步规范。成功举办了市八运会和国际小轮车比赛、国际象棋邀请赛、国际青少年乒乓球公开赛等赛事。169个社区布设了全民健身设施。人口与计划生育工作取得新突破，符合政策生育率达到91.6%，完成了省下达的人口计划指标。新建公共卫生项目12个，市县两级疾控中心建设基本完工。娄烦、阳曲两县15万人参加了农村新型合作医疗，参合率70.8%。社区卫生服务网络覆盖率68%。荣获“全国无偿献血先进城市”称号。社区换届圆满结束，全市社区调整为453个，新建34个“星光计划”项目，累计达到263个，180个社区达到“示范社区”标准。实施了重大建设项目监测制度，开通了统计信息网，统计监测服务水平不断提高。完成审计项目88个，审计决定落实率99.1%。外事工作进一步拓展，宗教、侨台、人防、气象、地震、档案、地方志、国防后备力量建设等工作和老龄、妇女、关心下一代等事业都取得了新成绩。

精神文明和民主法制建设不断加强。深入开展精神文明创建活动，加强未成年人思想道德建设，城乡文明程度进一步提高。各级政府自觉接受同级人大及其常委会的监督，积极支持政协开展工作，201件省市人大代表建议和476件省市政协提案全部办结。市政府一位副市长向市人大常委会述职。市政府提请市人大审议地方性法规5件，制订政府规章5件。积极开展法制宣传和法律援助，全民法制观念逐步增强。认真做好信访和人民调解工作，及时化解矛盾。加强社会治安综合治理，构建严打长效机制和治安防控体系，有力维护了社会稳定。整顿规范市场经济秩序，开展食品药品专项整治，加强物价监测调控，市场秩序逐步好转。

发展环境进一步优化。深化行政管理体制改革，市、县两级新一轮政府机构改革任务基本完成。认真贯彻《行政许可法》，又取消40项行政许可和65项非许可审批事项，保留的审批收费事项基本纳入两级政务大厅办理。“诚信太原”建设初见成效。建立了企业信用信息数据库，征集了12万户企业的信用信息。开展了“双百”试点企业创建活动，200户企业公开向社会作出诚信承诺。46个大中型市场实行了商品质量准入制度，实施面35%。50个社区开展了个人信用档案试点。全面推进“三项治理”，深入开展行风“双评”，坚决纠正部门和行业不正之风。

回顾一年来的工作，我们深切体会到，要把太原的事情办好，必须始终坚持以“三个代表”重要思想总揽全局，牢牢把握率先发展第一要务，努力实现更快更好的发展；必须始终坚持以科学发展观指导工作，按照“五个统筹”的要求，努力实现全面、协调、可持续发展；必须始终坚持贯彻中央宏观调控方针，落实省委、省政府决策部署，深化产业结构调整，走新型工业化道路，大力推进传统产业新型化和新兴产业规模化；必须始终坚持以改革促发展，敢于创新，勇于突破，用改革的办法解决前进中遇到的问题；必须始终坚持立党为公、执政为民，把广大人民群众的根本利益放在首位，关心困难群众生产生活，不断提高人民生活水平；必须始终坚持科学行政、民主行政、依法行政，转变政府职能，提高办事效率，形成支持干事、能干成事的氛围，为经济社会发展营造良好环境。这些是率先发展中我们获得的基本经验，也是今后应长期坚持的指导思想和重要原则。

各位代表，过去一年所取得的成绩来之不易。这是省委、省政府和市委正确领导的结果，是市人大、市政协和各位代表、各位委员监督支持的结果，是全市人民团结奋斗的结果。在此，我代表市政府，向辛勤工作在各条战线的工人、农民、知识分子和各级干部，向大力支持政府工作的人大代表、政协委员、各民主党派、工商联、人民团体、无党派人士，向关心帮助太原发展的国家、省驻并单位和驻并部队、武警官兵，向积极参与太原建设的港澳台同胞、海外侨胞和国际友人，表示衷心的感谢！

在肯定成绩的同时，我们也清醒地看到，我市经济社会发展中仍存在一些矛盾和问题。主要是：影响经济社会发展的深层次体制性、机制性障碍还较多，深化改革的任务还很重；具有省会城市特色的产业结构尚未形成，传统产业新型化、新兴产业规模化仍需大力推进；城乡发展不够协调，“三农”问题比较突出；城市承载能力与省会城市地位不相适应，城市规划、建设、管理水平有待提升；实现可持续发展的任务仍然艰巨，保护环境、改善生态、节约资源、安全生产需要下更大的力气；教育、文化、卫生、体育等公共产品和公共服务还不能满足人民日益增长的需要，社会事业发展还要付出更多努力；一些部门和工作人员的服务水平、办事效率与群众的要求还有差距，政府职能转变需要进一步加快。对这些问题，我们要在今后的工作中积极采取措施，认真加以解决。

二、2005年政府工作的总体要求和预期目标

2005年是全面完成“十五”计划的最后一年，也是建设中西部经济强市和生态园林城市的关键一年。做好今年的工作，具有十分重要的意义。当前，我们面临着很多加快发展的机遇。从发展的环境看，国家宏观调控取得阶段性成果，国民经济保持平稳运行，但资源紧张的状况短期内难以有效缓解，这为我们发挥资源优势、加快经济发展创造了有利条件。从发展的趋势看，从2002年开始，我市经济运行进入了新一轮经济周期的上升阶段，今年仍将处于快速增长的平台期。从发展的后劲看，通过几年结构调整，传统产业升级步伐加快，新兴产业正在发展壮大，经济增长方式逐步转变，自主发展能力不断增强。从发展的任务看，实现建设中西部经济强市的战略目标，最紧迫、最重要的仍然是继续保持更快更好地发展，迅速做大经济总量，进一步提高经济增长的质量和效益。我们要准确把握形势，明确肩负的责任，始终保持奋发有为的精神状态、埋头苦干的发展劲头、求真务实的工作作风，抢抓机遇，加速前进，千方百计保持经济社会持续健康协调发展的良好势头。

今年政府工作的总体要求是：以邓小平理论和“三个代表”重要思想为指导，深入贯彻党的十六大、十六届三中、四中全会和市委八届九次全会精神，用科学发展观统领经济社会发展全局，紧紧扭住全面率先发展第一要务，着力推进产业结构调整，着力加强资源环境保护，着力转变经济增长方式，着力深化改革、扩大开放，着力提高人民生活水平，着力解决经济社会发展中的薄弱环节，构建稳定和谐的社会环境，增强城市综合竞争力和可持续发展能力，为建设中西部经济强市和生态园林城市奠定坚实基础。

主要预期目标是：地区生产总值755亿元，增长15%；规模以上工业增加值282亿元，增长20%；财政总收入142亿元，增长18%；固定资产投资358亿元（城镇口径），增长20%；社会消费品零售总额260亿元，增长15%；地区外贸出口总额30亿美元，增长15%；城镇居民人均可支配收入10475元，增长12%；农民人均纯收入4260元，增长10%；居民消费价格总水平上涨幅度控制在3%左右；城镇登记失业率控制在4%左右；人口自然增长率控制在4.5‰以内；大气、水体实现功能区达标，空气质量二级以上天数突破240天。在实际执行中，我们将力争超额完成这些目标。

三、2005年政府工作的主要任务

（一）深入推进结构调整，加快构建具有省会特色的产业体系

全力推进传统产业新型化和新兴产业规模化。以建设新型能源和工业基地为方向，以深化和提高为着力点，努力构建具有省会城市特色的多元化、集约化、规模化新型支柱产业体系。坚决淘汰落后产业。严格控制普钢、水泥、电解铝等高能耗、高污染行业的生产规模，关停取缔储量和生产能力低、不符合安全生产条件的小煤矿、5000吨以下的造纸厂、20吨以下小转炉、六城区以内的土竖窑和露天原煤发运站。按期完成焦化行业清理整顿任务。改造提升传统产业。支持太钢150万吨不锈钢、二电厂六期扩建、古交电厂坑口电站、重型汽车、双喜全钢子午胎等重点项目建设，促进蓝星TDI、新东方铝业等项目达产达效。发展壮大新兴产业。扶持富士康、长城光电、亚日电脑等一批电子信息项目，发展铝镁合金、磁性材料、纳米材料等一批新材料项目，壮大宏全、三九啤酒、亚宝、瑞丰、瑞福莱等一批食品医药企业，努力打造特色明显、优势突出的新兴产业集群。重点加大对富士康项目的支持力度，完善配套工程和基础设施，确保二期工程建成投产，三期工程开工建设，工业产值达到25亿元以上。

大力发展园区经济。高新区要发挥科技创新优势，加快技术成果转化，新入区企业300家以上，孵化企业50家以上，力争引进几家世界500强、一批外资企业和国内大企业，成为培育壮大高新技术产业的重要基地。经济区要围绕入区企业和重点项目，抓配套、抓服务、抓规模，实现集约式发展，建成新兴产业规模化的示范园区，工业增加值达到10亿元以上，财政收入力争突破1亿元。民营区要拓宽发展空间，培育产业特色，聚集一批有实力的民营企业，办成以非公有制经济为主体的新兴产业基地。不锈钢工业园要理顺管理体制，完善园区规划，加强基础设施建设，落实优惠政策，积极引进上档次、有实力的大企业，力争入园企业达到35户，加工能力突破10万吨。

放手发展非公有制经济。清除政策性、体制性障碍，鼓励、支持和引导非公有制经济加快发展。推进产权制度改革，把一些国有、集体企业直接改造为民营企业。加大扶持力度，提高服务水平，支持民营骨干企业发展，培育几家销售收入10亿元以上的大型民营企业集团。非公有制经济在全市经济中的比重再提高2—3个百分点，达到40%以上。

加快发展县域经济。完善县域经济综合评价考核体系，建立促进县域经济发展的激励机制，探索制定对县域经济的扶持办法。坚持特色立县、项目强县，上一批对县域经济发展有重大影响的项目，形成新的产业特色和经济增长点。创新机制，加强管理，进一步精简压缩人员，提高县区执政能力和办事能力，争取更多的县区进入全省10强县行列。

（二）加大对“三农”的支持力度，不断改善农村的生产生活条件

继续狠抓支农政策的落实。顺应我国总体上已进入以工促农、以城带乡的发展阶段这一趋势，更加积极地支持“三农”。全面落实中央、省支持农业的各项政策，已实行的政策不打折扣、必须兑现，已给农民的实惠只能增加、不能减少。实施农村劳动力转移就业“阳光培训工程”，引导性培训5万人，职业技能培训1.2万人。加大扶贫开发力度，再有2.8万农村贫困人口实现脱贫。开展关爱革命老区建设活动。结合小城镇建设，积极引导乡镇企业改革创新，乡镇企业增加值和上缴税金分别增长25%和27%以上。

深入推进农业结构调整。大力发展具有城市农业特点的精品农业、高效农业、生态农业和特色农业，提高农业综合生产能力。重点扶持优质粮、果蔬、畜牧、苗木花卉、中药材、饲草、瓜果等七大优势产业，农村经济总收入380亿元，增长15%以上。严格耕地保护政策，继续抓好粮食生产，完成省下达的5亿斤粮食生产任务。菜、肉、奶、蛋产量分别达到135万吨、6.3万吨、9万吨和4.3万吨。推进农业产业化经营，抓好醋业、奶业和肉制品、果菜、粮油加工业，扶持水塔陈醋、森泰牧业、宏明养殖、旭光马铃薯等一批龙头企业，农产品加工园入区企业达到15家，农业产业化企业销售收入38亿元，增长15%以上。

加强农村基础设施建设。抓好农村主要经济通道、旅游路和断头路建设，改造5条县乡公路。高标准完成退耕还林10万亩、天然林保护工程6万亩、三北防护林建设5万亩，抓好汾河高效生态农业工程、东西山绿化、环城林带建设。新建农业节水园区3个、节水面积1.5万亩。建成5条精品小流域，治理水土流失面积18万亩。完善林区道路、通讯设施，严格落实责任，防止森林火灾发生。

加快农村社会事业发展。重点解决农民关心的科技服务、子女上学、就医看病、社会保障等四大问题。深入开展科技下乡活动，实施科技入户示范工程，发挥好“服务农民热线”的作用，为农民提供先进适用农业技术，实用新技术覆盖率达到95%以上，科技对农业的贡献率达到46%以上。加快非贫困县乡镇卫生院改造。以被征地农民、进城务工农民、乡镇企业职工等为重点，扩大农村养老保险覆盖面，新增参保人数6000人。全面试行农村最低生活保障制度。

（三）强化城市建设管理，不断改善人居环境

搞好城市规划建设。完成总体规划的修编工作，做好汾河治理美化二期、长风东大街、“城中村”改造修建性详细规划和绿线控制性规划。加快续建工程进度，滨河西路南北延伸、西北环连接线、城南防洪排水一期、垃圾焚烧发电、北郊污水厂扩建等重点工程要按期完工。抓好长风东大街、大东关两个出口路、城南污水厂、侯村垃圾填埋场及南堰垃圾中转站、采煤沉陷综合治理等重点工程建设，完成新建路、并州路、解放路、府西街等主要道路的大修任务。启动天然气工程。建设公路主枢纽客运站。旧城改造和经济适用房开复工100万平方米，竣工30万平方米。住房公积金归集累计突破65亿元，缴存职工突破80万人，个贷率达到18%。合理把握城市化进程，有效引导城镇化健康发展，建立逐步改变城乡二元结构的机制。以试点村为重点，妥善处理城乡关系，注意保护和节约土地，大力推进“城中村”改造。完成50条小街小巷的综合整治，新改建一批公厕。加大土地市场化运作力度，增加国有土地收益。

提升绿化美化水平。深入开展创建国家园林城市活动，实施汾河蓄水美化二期工程，抓好晋阳湖开发和学府公园、玉门河公园建设，提高迎泽大街、长风大街、迎宾路的绿化档次。出台《城市绿线管理办法》，建立“绿色图章”审批制度。大力开展全民义务植树活动和园林化单位、社区创建活动。按照“责权统一、重心下移”的原则，构建市、区、街、居四级分工明确、各负其责的城市管理体系。强化综合执法。提升12319城建热线的服务水平。以城乡结合部、城中村为重点，完善环境卫生长效管理机制，切实解决脏乱差问题。

加强资源环境保护。立足我市资源环境和产业结构的实际，从节约资源中求发展，从保护环境中求发展，从发展循环经济中求发展。加快工业污染源全面达标工作，一电、二电、太钢、西山国庆前完成重点限期治理工程。严格实施污染物排放总量控制，严把项目“三同时”审批关，从源头上防止新的污染。建立循环经济综合评价指标体系，抓好太钢、煤气化和经济区三个循环经济试点。落实清洁生产促进法，引导企业使用清洁生产工艺，树立一批清洁生产示范企业。抓好集中供热、煤改气工程和清洁燃料的推广使用，集中供热力争扩网700万平方米，无燃煤区扩大到14平方公里。加强扬尘污染和机动车尾气污染防治，努力改善空气质量。继续开展环保整治行动，强化环境监控和执法检查，严厉查处环保违法行为，三项主要污染物指标平均下降15%，努力创建山西省环境保护模范城。增强全民节约意识，大力节约能源和重要资源，降低单位能耗和用水量，建设节约型社会。加强水资源保护，继续加大关井压采力度。

（四）扩大对内对外开放，完善城乡市场体系

提高消费对经济的拉动作用。正确处理投资和消费的关系，提高城乡居民的消费能力，努力培育住房、汽车、教育、健康、旅游等消费热点。搞好城市商业体系和农村商业网点建设规划。支持华宇、美特好、唐久、田和等连锁企业改造升级和规模扩张，积极引进沃尔玛、赛特等国内外知名流通企业，提升商贸服务业总体水平。加快桥西农副产品批发市场、唐久超市物流园区等重点工程建设，努力构建高效开放的现代物流体系。支持零散小商铺升级改造，纳入便利店管理网络。积极开拓农村市场，建设2个大型农产品批发市场，发挥供销合作社特别是各类专业合作社的网络作用。搞好“农改超”试点，发展标准化农产品超市。强化对生活必需品等重要商品的市场监管和调控，建立防范市场异常波动的应急机制。

加大招商引资力度。依托高新区、经济区，开展富士康工业园配套项目和电子信息、新材料、食品医药项目招商；依托不锈钢工业园，开展不锈钢加工和综合配套项目招商。加快建设招商引资网站，开展网上招商和网上洽谈。组织企业参加大型经贸洽谈活动，吸引世界500强和跨国公司来太原投资。拓展外事、侨台工作领域，扩大同国际、国内友城的交往和交流，力争洽谈签约一批优势互补性强的大项目。加强与国家有关部门的联系，争取几个双多边受援项目。狠抓签约项目的落实和督查，提高合同履约率、资金到位率和项目成功率。

进一步扩大外贸出口。实施出口多元化战略，支持符合条件的企业申请进出口经营权。巩固煤焦、铝锭、金属镁等传统出口商品，扩大机电产品、高新技术产品的出口比重。鼓励企业参加各类出口交易会，进行质量、环境和产品认证，提高进入国际市场的能力。引导有实力的企业开展省外、境外投资，着力开拓东南亚、非洲、南美和东欧等地区的投资领域。

（五）大力深化各项改革，增强经济社会发展的内在活力

深化财政管理体制改革。以提高效益为核心，构建预算编制、执行、监督相分离的现代预算管理体制。50%以上的市级单位纳入国库集中支付试点。扩大政府采购范围和规模，把政府性基建投资、城市维修等列入政府采购。强化财政资金绩效评价，提高执政理财能力。全面推行行政事业性收费公示制度，取消不合理过渡。完善财税体制，科学合理地划分收支，构建各级财政自我发展、自求平衡的激励机制。加快“金财工程”建设，实现依法理财、科学理财和民主理财。加强财源建设，挖掘增收潜力，强化征管措施，确保应收尽收。完善公共财政框架，优化支出结构，合理安排各项重点支出，坚决控制、压缩一般性支出。

深化国有企业改革。完善国有资产监督管理体制和监管方式，建立国有资产经营责任、产权交易监督管理等规章制度，促进国有资产保值增值。通过产权转让、增资扩股、破产拍卖、收购兼并、内引外联等多种形式，规范推进全市国有企业改革重组。加快分离企业办社会职能和辅业转制，增强企业核心竞争力。积极稳妥地推动下岗职工出中心，着力解决协议期满滞留中心人员、已实现相对稳定就业人员和停产企业滞留中心人员的出中心问题。

深化投融资体制改革。落实企业投资自主权，对不使用政府投资建设的项目实行核准制和备案制，一律不再审批。出台《太原市产业投资指导目录》，加强对企业投资行为的引导。深化城建投融资体制改革和产权制度改革，实行国有资产和经营权的有偿转让，积极推行BOT、TOT等运作方式，真正做成1—2个项目，促进基础设施投资主体多元化。推进政府投资管理体制改革，严格执行项目公示制度、公开招投标制度和责任追究制度，加强资金监管，提高投资效益。加快建设系统改革步伐，企业脱钩，事业改制，市场放开，努力实现市政、园林、环卫、公交、供热、自来水、污水处理等公用事业的市场化运营。

深化事业单位体制改革。加快事业单位人事制度、分配制度、产权制度、社会保障制度改革，理顺事业单位编制、财政供养体制，基本完成事业单位改革任务。大力推进科技、教育、文化、卫生、体育等社会事业体制机制创新，优化结构，整合资源，增强活力。抓好高层次和紧缺人才的培养、引进和使用，加大留学回国人员服务工作力度，尽快建立门类齐全、素质优良、梯次合理、新老衔接的人才队伍。

（六）大力发展社会事业，推动社会全面进步

实施科教兴市战略。搞好制造业信息化、清洁能源行动、镁及镁合金基地三个试点建设。培育科技市场体系，创建科技成果转化中介服务平台。开展“科技成果转化突破年”活动，抓好100项重大科技成果应用转化示范项目，应用转化1000项科技成果，实施20个重大科技成果转化专项，创建20个科技成果转化示范单位。高新技术产业增加值占全市工业增加值的比重提高3个百分点，科技进步对经济增长的贡献率提高3个百分点。

加快教育事业发展。推进教育资源整合和布局调整，减少薄弱学校，再创建优质小学68所，优质初中32所，优质小学和初中的比例分别达到36%和50%。做好企业中小学剥离工作，并按新体制稳定运行。完成太原教育城域网和教育资源库基础工程建设，中学和小学信息技术开课率分别达到90%和70%以上。加强职业技术教育，毕业生合格率95%以上。抓好太原大学等市属高校建设，提高总体办学水平。搞好省城大专院校和优质高中新校址基础设施建设，引进各类院校发展特色教育。

完善公共卫生体系。抓好重大疾病和各类传染病的监测防控，建立公共卫生服务的长效机制。完成县级医院传染病区改造和市急救中心指挥大厅建设。统筹安排全市急救资源，建立市急救中心、县区急救站、医院急诊科、乡镇卫生院四级急救网络，急救半径控制在5—10公里以内。完善社区卫生服务体系，社区卫生服务网络覆盖率达到80%以上。强化人口与计划生育工作，稳定低生育水平，提高人口素质，出生率控制在9‰以下，全面兑现农村计划生育家庭奖励政策，努力创建省会城市计划生育工作先进城市。

发展文化体育事业。实施精品工程，弘扬晋阳文化，办好山西省第四届广场文化艺术节。加强文化基础设施建设，适时启动专题博物馆、档案馆、音乐厅项目。抓好广电、报业、演艺、电影的市场化运作，发展壮大文化产业。完成城区有线电视数字化整体平移工作，完善农村广播电视设施。大力发展竞技体育，积极申办国际国内体育赛事。改善体育场馆条件，在全市社区布设和完善全民健身设施。搞好全民健身活动中心、汾河公园体育设施和游泳跳水馆等项目。以申报全国文明城市为契机，全面落实《公民道德建设实施纲要》，加强和改进未成年人思想道德建设，深入开展精神文明创建活动。

不断壮大旅游产业。打造省城旅游品牌，着力构建以文物景点、文化欣赏、生态观光、红色旅游为主的旅游产业新格局。以建设旅游中心城市为切入点，完善旅游基础设施，深入开展晋祠、天龙山等景区的综合整治，保护好历史文化遗迹和街区，完成汾河二库、五龙山生态观光园等旅游景区规划，办好太原国际面食节、太原旅游交易会等文化节会。加强旅游市场监管，提高宾馆、旅行社的服务水平。全年接待海内外游客1450万人次，旅游总收入增长13%以上。

提高社区建设水平。推进社区服务网络化和信息化建设，社区普遍成立服务中心，不断提高社区家政服务、文化娱乐、托幼养老、环境整治的服务质量，促进社区服务业向社会化、产业化、规模化方向发展。加强社区管理服务队伍建设，提高社区干部生活待遇。

加强国防后备力量建设，落实优抚安置政策，增进军政军民团结，继续创建“双拥模范城”。认真组织实施第一次全国经济普查和人口抽样调查，加大对经济社会运行的监测力度。做好“十一五”规划和2020年远景规划的编制工作。人防、气象、地震、档案、地方志等各项工作和老龄、妇女、关心下一代等各项事业，在新的一年都要实现新发展，取得新成绩。

（七）增强解决群众生产生活问题的能力，不断提高人民生活水平

做好就业和再就业工作。多渠道开发就业岗位，加大差额购买、补贴购买公益岗位的力度。充分发挥再就业服务平台作用，组织开发一批有一定收益的服务项目，安置就业困难人员。认真落实再就业扶持政策，支持下岗失业人员自主创业。全年下岗失业再就业4.3万人，其中安置“4050”人员5000人。抓好残疾人就业工作，就业保障金突破3000万元，切实发挥对残疾人就业的保障和促进作用。

完善社会保障体系。积极推进机关事业单位养老保险制度改革，将全市机关事业单位的合同制人员纳入养老保险范畴。完善灵活就业人员社会保险参保和接续办法，做好中央企业、非公有制企业医疗保险参保工作，扩大社会保障覆盖面，企业养老、失业、医疗保险参保人数分别达到57.5万、67.5万和75万人，进一步加大基金征缴清欠力度。做好企业退休人员社会化管理工作，社会化管理服务率达到90%以上，其中社区管理服务率达到70%以上。

关心困难群众生产生活。严格执行国企改革的政策规定，维护职工的合法权益。进一步规范农村土地征用和城镇拆迁行为，落实补偿标准，依法文明办事。加大清理拖欠工程款力度，按期完成清欠任务。建立企业工资保障金制度，形成防止拖欠农民工工资的长效机制。实施义务教育控辍保学和爱心助学工程，保证贫困家庭和农民工子女接受教育的权利。严格实行义务教育“一费制”，治理教育乱收费。建立农村医疗救助制度。加强医疗服务市场监管，严格管理药品价格和医疗检查收费。逐步建立以低保制度为支撑，医疗救助、教育救助、灾害救助、农村五保供养、特困户救济为补充，社会福利和慈善事业相衔接，覆盖城乡的新型救助体系。

各位代表，维护好、实现好、发展好广大人民群众的根本利益，是政府一切工作的出发点和归宿。新的一年，市政府要为全市人民办好十件实事：一是继续全部免征农业税和农业税附加，对种粮农民实行直接补贴。二是实施农村饮水解困工程，再解决6万农村人口饮水困难和水质不达标问题。三是启动娄烦库区移民工程，对8000名农村贫困人口实施移民搬迁。四是完成4.4万平方米农村中小学危房改造，免费为4.5万名义务教育阶段特困生提供教科书，免除9700名特困生的学杂费。五是建设康居工程住房100万平方米，落实200户廉租住房。六是城市绿化覆盖率和绿地率提高1个百分点，人均绿地增加0.5平方米。七是推进农村新型合作医疗，阳曲、娄烦和迎泽、杏花岭、尖草坪、万柏林四个城区农民参合率达到80%以上，覆盖农村人口36万。八是新增城镇就业人数12.6万人，转移农村富余劳动力3万人。九是全面落实最低工资制度，维护企业职工合法权益。十是再完成村村通油路工程160公里，实施村村通班车工程，90%以上的行政村通班车。十件实事关系人民群众的切身利益，我们一定要扎扎实实把实事办好。

（八）切实维护社会稳定，努力营造安定和谐的社会环境

落实维护稳定责任制。做好群众来信来访工作，畅通社情民意反映渠道。加强矛盾排查和人民调解工作，化解矛盾纠纷，努力把不稳定因素解决在基层，消除在萌芽状态。按照“分级负责、归口办理”的原则，严格信访工作责任制，开展信访突出问题和群体性事件的专项治理，落实领导包案制度，着力解决重点疑难信访案件，切实减少越级上访、集体上访。健全应对突发事件的组织体制和应急机制，形成统一指挥、反应灵敏、协调有序、运转高效的预警体系，对各类突发事件迅速反应、果断处置。落实民族、宗教政策，维护社会政治稳定。

加强社会治安综合治理。深入推进严打整治斗争，依法严厉打击各类犯罪活动，尤其要重点打击群众反响强烈的入室盗窃、沿路抢夺等犯罪行为。坚持不懈地开展同“法轮功”等邪教组织的斗争，坚决扫除黄赌毒等社会丑恶现象。大力开展普法教育，拓展规范法律服务和法律援助。完善“条块结合、以块为主”的社会治安防控体系，开展基层安全和平安县区创建活动，逐步形成具有省会特点的社会治安综合治理格局，努力进入全国治安状况最好城市行列。

加大市场监管力度。以质量监督、药品监督、价格监管和食品卫生安全为重点，大力整顿和规范市场经济秩序。对集贸市场和加油站等经营场所实行专项治理，对食品、药品、农资、汽车配件和医疗器械、建材等商品实行专项打假，对出版物、网吧及娱乐场所等文化市场秩序实行专项整顿，对增值税发票犯罪等破坏税收秩序的行为进行专项打击，对各类非法传销组织依法予以严惩。坚决制止对企业的“五乱”行为，创造良好的经营环境。

切实抓好安全生产。始终坚持“安全第一、预防为主”的方针，严格落实安全生产责任制，健全安全监管体系，强化安全执法和考核，加大事故责任追究力度。突出抓好煤矿、非煤矿山、危险化学品等行业的安全整治和交通安全、消防安全，防止重特大事故发生，实现安全生产形势稳定好转，切实保护好人民群众的生命财产安全。

（九）提高政府行政能力和水平，不断创优发展环境

坚持科学决策。建立和完善群众参与、专家咨询、集体决策相结合的决策机制和制度，健全决策规则，规范决策程序，强化决策责任，减少决策失误。凡涉及经济社会发展全局的重大决策，必须由领导班子集体做出决定；凡专业性、技术性强的决策，都要请专家评估和论证；凡涉及人民群众切身利益的决策，都要举行听证和公示。建立决策后的评价制度和决策责任追究制度，健全决策讲科学、执行讲效率、监督讲实效的各项措施。

坚持民主监督。定期向人大及其常委会报告工作，自觉接受政协的民主监督，认真听取各民主党派、工商联、无党派人士和人民团体的意见。积极办理人大代表建议和政协提案。推进电子政务建设，建立新闻发布制度和发言人制度，扩大政务公开范围，能够公开的都要向社会公开，提高行政透明度。加强基层民主建设，完善村民自治、社区自治和职工代表大会制度，继续推进村务公开、厂务公开，依法保障群众的知情权、参与权、选择权和监督权。

坚持依法行政。深入贯彻《行政许可法》，进一步清理、精简行政许可事项，完善政务大厅功能，规范审批行为，提高审批效率。严格规范备案和核准的范围和程序，对已经取消的和下放的审批事项绝不能以备案的名义搞变相审批和权力上收，新设定的行政许可事项必须于法有据。推进政府法制建设，规范行政执法行为，建立行政执法评议考核制、执法过错责任追究制、执法人员资格认证制，出台公务员行政奖惩实施办法、行政投诉管理办法，做到有权必有责，用权受监督，侵权要赔偿，违法要追究。

转变政府职能。以“保持共产党员先进性教育活动”为契机，进一步加强政风建设，认真解决在思想、组织、作风以及工作方面存在的突出问题，做到教育活动与各项工作“两不误、两促进”，让群众切实感受到先进性教育活动的成效，使各级政府和广大公务员始终成为人民利益的忠实维护者。加快政企分开、政事分开、政社分开，切实把政府职能转到经济调节、市场监管、社会管理和公共服务上来。创新管理体制和运行机制，改进工作作风，提高办事效率。大力精简会议和文件，腾出更多的时间和精力，深入基层、深入群众、深入困难多、问题多的地方，帮助基层解决问题，帮助群众解决困难。勤于学习，善于思考，勇于创新，努力提高科学判断形势的能力、驾驭市场经济的能力、应对复杂局面的能力、总揽全局的能力和解决实际问题的能力。坚持从严治政，严格执行反腐倡廉的各项规定，全面落实党风廉政建设责任制，积极开展行政效能监察，深化民主评议政风、行风活动，强化审计监督，从源头上预防和治理腐败，树立勤政、务实、高效、廉洁的政府形象。

创优发展环境。推动以“诚信太原”为主题的优化发展环境工作向高层次、宽领域延伸，创造公开透明、优质高效的政务环境，统一开放、公平公正的市场环境，重诺守信、有诺必践的人文环境，努力把太原建成文明开放、创新创业、富有吸引力和竞争力的现代城市。提高公务员素质，健全公务员信用记录制度，规范公务员守信行为。建立全市统一的企业信息分析、评价和发布平台，继续开展“诚信企业”创建活动。加强市民诚信建设，扩大社区建立市民诚信档案的范围。

各位代表，新的目标鼓舞人心，新的事业催人奋进。让我们在“三个代表”重要思想指引下，认真贯彻党的十六届三中、四中全会精神，按照省委、省政府和市委的决策部署，全面落实科学发展观，开拓进取，团结拼搏，圆满实现“十五”计划目标，为建设中西部经济强市、生态园林城市而努力奋斗！
